# Evaluation of Intrinsic Image Algorithms to Detect the Shadows Cast by Static Objects Outdoors

**DOI:** 10.3390/sl21013333

**Published:** 2012-10-01

**Authors:** Cesar Isaza, Joaquín Salas, Bogdan Raducanu

**Affiliations:** 1 CICATA Qro., Instituto Politécnico Nacional, Cerro Blanco 141, Col. Colinas del Cimatario, Santiago de Queretaro, C.P. 76090, Mexico; E-Mail: jsalasr@ipn.mx; 2 Computer Vision Center, UAB Campus, Bellaterra 08193, Barcelona, Spain; E-Mail: bogdan@cvc.uab.es

**Keywords:** video sequences, shadow detection, intrinsic images, illumination component

## Abstract

In some automatic scene analysis applications, the presence of shadows becomes a nuisance that is necessary to deal with. As a consequence, a preliminary stage in many computer vision algorithms is to attenuate their effect. In this paper, we focus our attention on the detection of shadows cast by static objects outdoors, as the scene is viewed for extended periods of time (days, weeks) from a fixed camera and considering daylight intervals where the main source of light is the sun. In this context, we report two contributions. First, we introduce the use of synthetic images for which ground truth can be generated automatically, avoiding the tedious effort of manual annotation. Secondly, we report a novel application of the intrinsic image concept to the automatic detection of shadows cast by static objects in outdoors. We make both a quantitative and a qualitative evaluation of several algorithms based on this image representation. For the quantitative evaluation, we used the synthetic data set, while for the qualitative evaluation we used both data sets. Our experimental results show that the evaluated methods can partially solve the problem of shadow detection.

## 1. Introduction

A shadow is the result of an opaque object obstructing light which otherwise would directly illuminate a surface. Shadows are present in almost every computer vision application, where they may give rise to undesired effects in methods including segmentation [1,2], recognition [3], and tracking [4–6]. The main problem is that their existence may alter our interpretation of the scene, making our models drift—sometimes up to the point of failure. Consequently, it is desirable to detect them and to attenuate as much as possible their negative effects [7,8]. However, in some situations their presence is attractive as they may help to obtain 3D information for scene reconstruction [9,10], for instance.

The general problem of shadow detection can be classified depending on whether the objects casting the shadow are static [11,12] or moving [13,14]. However, a very important factor when considering this categorization is the scale of time. For instance, in outdoors the shadows cast by objects such as buildings, lamp posts, and trees during daylight can be interpreted as static if we consider in our analysis a temporal window of a few seconds. In this case, no significant changes will be perceived in the scene and existing techniques for moving cast shadow detection [15] cannot be applied. On the other hand, if we consider a temporal window of a few hours, the same shadow could be interpreted as a moving object. Although the problem of detecting moving shadows has been extensively studied [13,16], the problem of detecting static shadows outdoors, over long periods of time, such as days, has received little attention [17].

Our paper presents two main contributions. First, we introduce the use of synthetic images for which ground truth can be generated automatically, avoiding the tedious effort of manual annotation. In the process, we generated a custom database based on two image data sets (these data sets are publicly available for download at http://imagenes.cicataqro.ipn.mx/shadows/), one real and one synthetic. The real data set was acquired outdoors during several days using a fixed camera, which was overlooking a quiet area without moving objects. The synthetic data set was created using a rendering software. Secondly, we perform a quantitative and a qualitative evaluation of several algorithms for shadow detection based on the intrinsic image (the concept of intrinsic images was introduced by Barrow and Tenenbaum [18] as a way to describe an image in terms of characteristics such as range, orientation, reflectance, color, texture, and incident light) representation [19–22], all of which uses the reflectance component. The quantitative evaluation was done for the synthetic data set, while the qualitative one was done with both data sets.

The rest of the paper is organized as follows. In Section 2, we present a survey of the existing research literature about shadow detection algorithms. Then in Section 3, we describe the synthetic and real data sets used in our evaluation. In Section 4, we recall the definition of intrinsic images and then present the algorithms used in our evaluation study. In Section 5, we present quantitative and qualitative results to compare the methods. Finally, Section 6 contains our conclusion and our ideas for future work.

## 2. Related Work

In this section, we summarize work in shadow detection. For a clear presentation, we distinguish between static and moving shadows.

### 2.1. Static Shadow Detection

Static shadows give us clues about the scene represented in an image or a video sequence, including the shape, the objects, and the relative position of the light sources [23]. Additionally, these shadows seem to modify the perceived shape and color of the objects [24]. Some methods have exploited the color properties of objects as they are affected by shadows. For instance, Nagao *et al.* [11] and Scanlan [25] used histogram analysis to detect shadows. On the other hand, Jiang and Ward [26] reported a method for identifying and classifying shadows in real images based on the constraints that shadows possess in both intensity and geometry. Suzuki *et al.* [27] proposed a compensation method to remove shadows in aerial images transforming the red, green, and blue (RGB) values of the original image into hue, saturation and intensity (HSI) values. Some other researchers have exploited the relationship between the different homogeneous color regions in an image. This allows them to obtain first the edges of a shadow and then extract the shadow area. In addition, several color spaces have been explored in order to detect shadows, e.g., normalized red, green, and blue (rgb), hue (H) and saturation (S), *c1c2c3* and *l1l2l3* [28]. Finlayson *et al.* [29] proposed a method to process a 3-band color image to remove the shadows based on edge information. Moreover, Gevers and Stokman [30] also used color information to classify edges based on whether the transition between regions is due to shadows, abrupt surface orientation changes, illumination, or material changes. Levine and Bhattacharyya [31] developed a strategy that does not require camera calibration or other a priori information regarding the scene.

### 2.2. Moving Shadow Detection

Unlike static shadows, the moving ones are associated with dynamic objects. In many situations, moving objects and their shadows are detected as one single region. The above effect may require a stage to separate the object from its cast shadows, like the one described in the method proposed by Sonoda and Ogata [32], which is based on projective geometry. In addition, other authors proposed different strategies to detect moving shadows based on the use of diverse color models. For example, Horprasert *et al.* [33] used a brightness and chromaticity color model. Moreover, Mikic *et al.* [34] used a method that combines different color spaces. The authors considered three features at each pixel: brightness, normalized red and normalized blue. In this method, each feature is analyzed by a posterior probability estimator that computes probabilities for three classes: background, foreground and shadows. Nadimi and Bhanu [35] proposed an algorithm to detect moving shadows in outdoor environments based on a spatio-temporal albedo test and a dichromatic reflection model. In addition to the color spaces, some shadow features such as transparency (a shadow always darkens the region upon which it falls) and homogeneity (the relationship between pixels under shadows is roughly linear) have been used to detect moving shadows in outdoor traffic scenes [36]. However, this method considers a linear relationship between shadow and non-shadow regions and only gray-scale images are processed. On the contrary, Cucchiara *et al.* [37] developed a method for segmenting moving objects without their shadows by using color information. This algorithm transformed the original input image from RGB color space to hue, saturation, and value (HSV). Recently, Sanin *et al.* [15] presented a survey of several techniques for moving cast shadow detection. It is important to understand that the strategies these authors compared cannot be applied to the problem that we describe, because these techniques first detect changes in the scene (moving objects) and then classify the detected pixels as foreground (object) or shadow. Under the above assumption, the shadows cast by static objects will be part of the background; or, if a large enough interval of time is selected to perceive changes in the shape and position of the shadows cast by static objects, the moving shadow detection strategies will detect only the parts of the shadow that change, which is typically the region around the boundaries.

## 3. Data Sets

**Figure 1.**
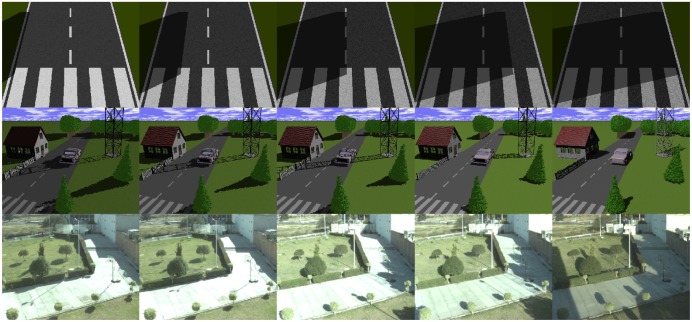
Data sets used for the evaluation of algorithms. The first and second rows are synthetic and the third row is a real scenario. The images illustrate changes in the illumination due to the relative movement of the sun.

For algorithm comparison, we used two data sets, one synthetic and one real (see Figure 1). The synthetic data set consists of two sequences and has been used for quantitative evaluation; meanwhile for the qualitative evaluation, we used real and synthetic sequences. To build the data sets, we considered only daytime, when the sun is by far the main source of light. It is important to mention that in scenarios lighted with other sources, such as fluorescent or street lights, the shadows cast by static objects will be always static and perhaps only changes in intensity will be perceived. In the next subsections we present details regarding the generation of these data sets.

### 3.1. Synthetic Data Set

A serious limitation in the systematic evaluation of algorithms to detect shadows cast by static objects during extended periods of time is the lack of a standard data set with annotated ground truth. Based on this fact, we used two synthetic sequences that simulate the changes in the sun's position over a long period of time (days) for a particular geographical position. The advantage of using synthetic images is that the ground truth is automatically generated.

**Figure 2.**
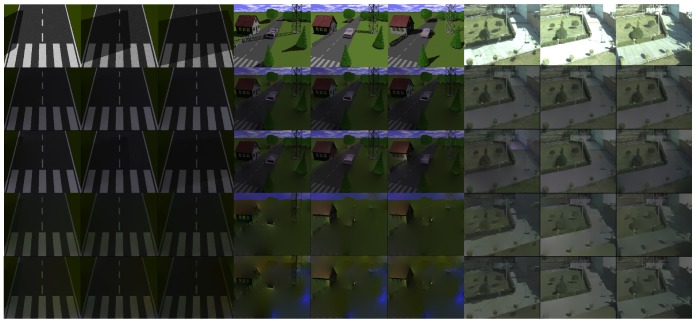
Reflectance images. Top, input images with different illumination conditions; second, third, fourth, and fifth rows are reflectance images computed with the Weiss [19], Matsushita *et al.* [20], gray [39] and color Retinex [40] methods respectively.

The synthetic sequences were rendered using the POV-Ray software [38]. We designed the first synthetic sequence in accordance to the one introduced by Masushita *et al.* in [20], to analyze the problem of the computation of intrinsic images. This data set has 20 frames with a resolution of 512 × 384 pixels (width × height). It represents white lines on a road surface (see first, second and third columns in Figure 2). The shadow effect was created using a rectangular object out of the field of view of the camera, changing the light source positions on the horizontal axis. The second synthetic sequence simulates the sun position over 12 consecutive days. We generated 40 frames per day with a resolution of 512 × 384 pixels (width × height). We designed this data set considering objects that exist in a typical outdoor location. We included in our scenario static objects such as buildings, a road, trees, and bushes, among others (see fourth, fifth and sixth columns in Figure 2).

In the synthetic sequences, the shadow effect was created using the following procedures. First, we computed the sun position in a given geographical location. We used an off-the-shelf Geographical Positioning System (GPS) to obtain the latitude, longitude, and elevation of a camera installed in the real outdoors scenario. Then, using the algorithm presented by Reda and Andreas [41] the sun position (elevation and azimuth angles at a given location) as a function of the local time and position of the observer was computed from dawn to dusk during twelve consecutive days. After that, the values of the elevation and azimuth angles were transformed to Cartesian coordinates for the rendering software to simulate the changes in the sun position.

### 3.2. Real Data Set

In addition to the synthetic sequences, we recorded a real data set. For this purpose, we positioned a fixed camera on the roof of a building and took images from dawn to dusk during seven consecutive days (see seventh, eighth, and ninth columns in Figure 2). The camera was fixed to capture a motionless area, to facilitate the analysis of shadows cast by static objects during this long time interval. Each image has a resolution of 1,032 × 776 pixels (width × height). We selected the location, based on the challenge that it represents for the detection of shadows cast by static objects. In the scenario, there are regions that are shaded during all daylight and others with huge shadows. Additionally, some shadows have significant changes in the intensity, up to the point that it is difficult to define the boundaries. This data set also has isolated shadows cast by trees and shadows mixed that are cast by several objects. Another important feature of the data set is that only small fast traveling clouds appear in the sequence, resulting in the presence of shadows during all daylight.

## 4. Evaluating Algorithms

Our evaluation considers the algorithms to derive intrinsic images introduced by Weiss [19], Matsushita *et al.* [20], Land and McCann [39], and Blake [21]. Because all of the algorithms are based on the concept of intrinsic images, we review it first. Then, we describe in some detail each of the algorithms evaluated. Lastly, because the algorithms were primarily created to compute intrinsic images, we illustrate how they could in principle be used to detect shadows.

### 4.1. Intrinsic Images

The concept of intrinsic images was introduced by Barrow and Tenenbaum [18]. It describes an image decomposition in terms of characteristics such as range, orientation, reflectance, and incident illumination. One of the simplest models is described by the product *I*(**x**, *t*) = *R*(**x**, *t*)*L*(**x**, *t*), where *I*(**x**, *t*) is an image, **x** is a pixel index, and *t* represents the frame index respect to time. The reflectance image *R*(**x**, *t*) represents the properties of the object to reflect light in the direction of the pixel **x**. The illumination *L*(**x**, *t*) describes the distribution of the incident light and accounts for some of the shading effects and shadows. Deriving this decomposition is a fundamentally ill-posed problem [42]. Weiss shows that this problem can be solved if one considers the reflectance to be constant while the illumination varies [19]. Matsushita *et al.* [20] extended Weiss' method to handle scenes where the Lambertian assumption does not hold. They consider that both the illumination and reflectance may change. In their method, they analyze the magnitude of the gradient in the reflectance component. If the magnitude in a given position is larger than a given threshold, the illumination values at that position are removed and added to the reflectance image. As a result, for each input image two others are obtained, one for the reflectance and another for the illumination. Although with this method a reflectance component is obtained representing the texture in the scene, some texture appears in the illumination image even for different values of the threshold. Another strategy to derive intrinsic images was introduced by Land and McCann [39]. They proposed the Retinex theory, which expresses that the reflectance component can be separated from the illumination if the later is assumed to vary slowly. An extension which uses color have been introduced by Finlayson [8,40]. In a different approach, a learning-based method to separate reflectance and illuminate was proposed by Tappen *et al.* [42]. They successfully separated the reflectance and the illumination components for a light source in a synthetic data set. In 2009, Grosse *et al.* [22] introduced an intrinsic image model with three terms: reflectance, illumination, and specularity *C*(**x**, *t*). All together, this decomposition is expressed as: *I*(**x**, *t*) = *R*(**x**, *t*)*L*(**x**, *t*) + *C*(**x**, *t*). Nonetheless, in this model, the problem of factoring the information between reflectance and illumination remains.

### 4.2. Weiss' Algorithm

Weiss [19] proposed that intrinsic components can be derived by using a sequence of images without motion acquired with a fixed camera in an outdoor scene under varying illumination conditions. This method uses the statistics of natural images [43] and assumes that illumination images will give rise to sparse filter outputs. Then, the scene reflectance image is obtained by taking the median of the filtered image sequence in the log domain. Additionally, the method assumes that the scene is Lambertian and the fact that illumination images have less contrast than reflectance images.

Weiss uses the following equation to derive intrinsic images:

(1) $${\left\{I(x,t)\right\}}_{t=1}^{T}=R(x){\left\{L(x,t)\right\}}_{t=1}^{T}$$

For convenience, Weiss worked in the log domain. In what follows, we will represent variables in the log domain using lower-case letters, e.g., *i*(**x**, *t*) to represent the logarithm of *I*(x, *t*). According to Weiss' method, a reflectance edge image is computed by taking the median along the time axis of the convolution between the derivative filter and a given image:

(2) $$r(x)=\text{median}[f_{m}*i(x,t)]$$

where *r*(**x**) is the constant edge reflectance image and *f_m_* represents the derivative niters *f*_0_ = [0, 1, −1] or *f*_1_ = [0, 1, −1]*^T^*. Then, the illumination edge images *l*(**x**, *t*) are computed subtracting the edge maps of the input and the reflectance images:

(3) l(x,t)=i(x,t)−r(x)

### 4.3. Matsushita et al.'s Algorithm

Matsushita *et al.* [20] analyzed the reflectance component based on the strategy proposed by Weiss and the derived time variable of reflectance and illumination images. They noticed that in real-world scenes, the Lambertian assumption is not sufficient to derive intrinsic images. Matsushita *et al.* analyzed the magnitude of the gradient of the reflectance edge image *r*(**x**) by assuming that texture information should not be present. Then, if the magnitude of the gradient in a given position of the reflectance image is larger than a given threshold *T*, the texture edge is removed from the illumination image *l*(**x**, *t*) and added to the time-varying reflectance image denote by

(4) $$r(x,t)=\{\begin{array}{l} r(x)+l(x,t)\;\text{if}\;\left|r(x)\right|>T\\ r(x)\;\text{otherwise} \end{array}\text{and}$$

(5) $$l(x,t)=\{\begin{array}{l} 0\;\text{if}\;\left|r(x)\right|>T\\ l(x,t)\;\text{otherwise} \end{array}$$

After the time-varying reflectance and illumination component edge maps are obtained, a deconvolution process is applied to get a reconstructed image, as

(6) $$<\hat{r},\hat{l}>=g*\left(\sum_{m}f_{m}^{\prime }<r,l>\right)$$

where *r̂* and *l̂* are the reflectance and illumination time-varying reconstructed images, $f_{m}^{\prime }$ is the inverse filter of *f_m_*, and *g* is the filter which satisfies the equation:

(7) $$g*\left(\sum_{m}f_{m}^{\prime }*f_{m}\right)=\delta$$

### 4.4. Gray Retinex Algorithm

The Retinex algorithm, proposed in [39], analyzes logarithm image gradients. This method considers that small gradients are due to changes in the illumination, while large gradients represent texture. The threshold value that classifies the edges into reflectance or illumination is defined for the horizontal *i_x_*(**x**, *t*) and vertical *i_y_*(**x**, *t*) derivatives. This method can be applied to gray-scale images or each color band separately. The formal description for gray-scale images is:

(8) $$r_{k}(x,t)=\{\begin{array}{l} i_{k}(x,t)\;\text{if}\;\left|i_{k}(x,t)\right|>T\\ 0\;\text{otherwise} \end{array}$$

where *k* can be either *x* or *y*.

### 4.5. Color Retinex

An extension of the Retinex algorithm to color images has been proposed by Finlayson *et al.* [40]. This method analyzes logarithm image gradients in color space. Here, two thresholds are considered, one for the chromaticity *T_C_* and another for the brightness *T_B_* subspace, as

(9) $$r_{k}(x,t)=\{\begin{array}{l} i_{k}^{B}(x,t)\;\text{if}\;\left|i_{k}^{B}(x,t)\right|>T_{B}\\ i_{k}^{C}(x,t)\;\text{if}\;\left|i_{k}^{C}(x,t)\right|>T_{C}\\ 0\;\text{otherwise} \end{array}$$

where *k* can be either *x* or *y*.

### 4.6. Shadow Detection

**Figure 3.**
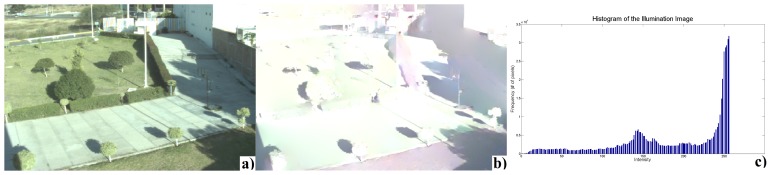
Example of an illumination histogram. (**a**) Input image; (**b**) illumination component computed with the method proposed by Matsushita *et al.*; (**c**) Corresponding histogram of the illumination with two Gaussian tendencies. The highest Gaussian tendency corresponds to non-shadow pixels.

None of the methods described earlier was designed to estimate the position of the shadows. However, the illumination component, *l*(**x**, *t*), encodes information about shadows, *S*(**x**, *t*). So to compare their usefulness for shadow detection, we devised a method based on thresholding the illumination histogram. Figure 3 illustrates an example of the original image, where illumination and its corresponding histogram are estimated with the method proposed by Matsushita *et al.* Then, each pixel of the illumination image is classified into shadow (*C*1) or non-shadow (*C*2). For this purpose, an experimental threshold (*T*) is selected and the shadow segmentation process is achieved, as

(10) $$S(x,t)=\{\begin{array}{l} C1\;\text{if}\;l(x,t)<T\\ C2\;\text{otherwise} \end{array}$$

We used the same procedure to detect shadows in all of the algorithms that were evaluated.

## 5. Experimental Results

The purpose of the experiments is twofold. On one hand, we show how the intrinsic image methods are used to detect shadows. On the other hand, we report quantitative and qualitative results based on the illumination images computed with the intrinsic derivation methods described in the previous section.

### 5.7. Intrinsic Image Results

To illustrate the results of the methods to derive intrinsic images, we selected three frames from each data set. In Figure 2, reflectance images computed with the evaluated algorithms are illustrated. The first row in this figure includes input images with shadows cast at different locations due to variations of the sun position. All of the columns of the second row show the reflectance image computed with the Weiss' method. Here, a static reflectance image for the respective sequence is obtained. In the third, fourth and fifth rows, reflectance images computed with the Matsushita *et al.*'s, gray and color Retinex methods are presented. The results show that shadow information is present in all reflectance images. Moreover, in the second and third rows of Figure 2, it may be seen that the reflectance images derived with the Weiss' and Matsushita *et al.*'s methods have more texture than the gray and color Retinex algorithms. Furthermore, it should be noted that the shadow detection process is based on the illumination component and not on the reflectance one. Thus, we selected the thresholds for all the methods to derive the intrinsic images based on the visual quality of the illumination component.

**Figure 4.**
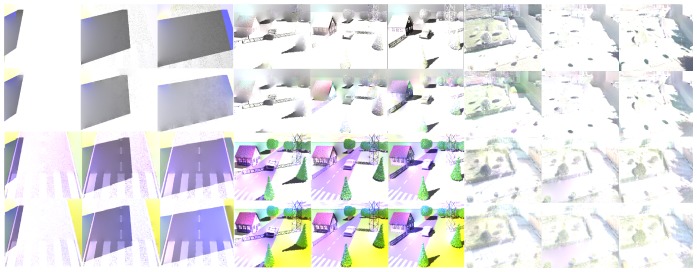
Corresponding illumination images of Figure 2. From top to bottom: Weiss [19], Matsushita *et al.* [20], gray [39], and color Retinex Methods [40].

Results in Figure 4 show that all illumination images contain texture information. In the frames computed using Weiss's method, the texture that represents the white lines is visible inside and outside of the shadow area, while in the frames obtained with Matshusita *et al.*'s method, the texture information is presented inside the shadow region. Also, a very smooth texture pattern appears outside the shadow region. Moreover, some boundaries of the shadow region estimated with the Matsushita *et al.*'s method become too diffuse as a result of the value of the threshold selected. This diffuse effect near the boundaries causes problems in the segmentation of shadows. In the illumination images computed with the gray and color Retinex algorithms, some purple and green color artifacts appear, because there is mixed information about gradients due to illumination and reflectance and one single threshold is not sufficient to separate both components.

### 5.2. Quantitative Analysis

**Figure 5.**
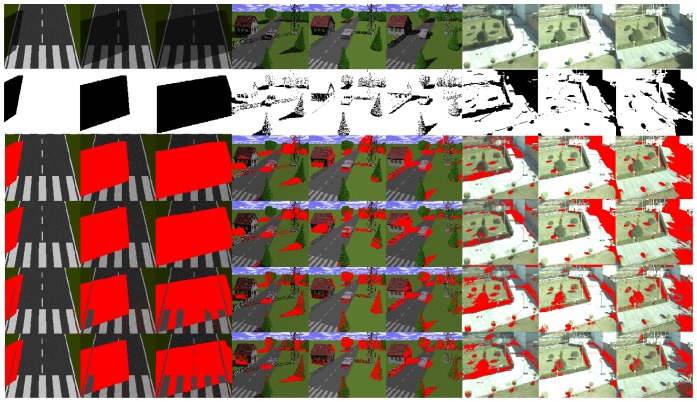
Qualitative results in the synthetic data set. The first and second rows are input and ground truth of shadows. The third, fourth, fifth, and sixth rows are the results of the Weiss [19], Matsushita *et al.* [20], gray [39], and color Retinex methods [40], respectively.

**Figure 6.**
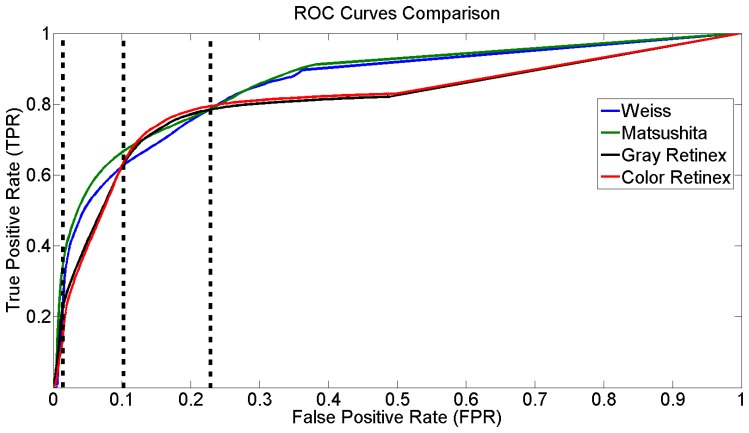
ROC Curves for the Weiss [19], Matsushita *et al.* [20], gray [39], and color Retinex methods [40]. The vertical lines define the four regions where the algorithms are compared.

In this section, a quantitative assessment of the methods to detect shadows cast by urban infrastructure, based on the derived illumination image, is presented. To evaluate the performance of the methods systematically, we used the Receiver Operating Characteristic (ROC) analysis. For our evaluation, we used forty synthetic images (samples of these images are illustrated in fourth, fifth and sixth rows in Figure 5). The ground truth was computed automatically by subtracting the shadows from the shadowless image. First, the intrinsic image derivation methods were used to compute the reflectance and the illumination component of each frame. Then, a segmentation process based on the histogram analysis was applied. The curves in Figure 6 represent the false positive shadow detection rate on the horizontal axis and the shadow detection rate on the vertical one.

In addition, different performance results can be obtained for the methods in relation to the measuring parameter. For example, if we consider the value measured at the point in the ROC space that is located at the northwest point, the result would show that the best method to detect shadows based on the derived illumination image is color Retinex. Nonetheless, if we selected the area under the curve as a measurement parameter [44], the best method is that of Matsushita *et al*.

For a better analysis of Figure 6, we can consider the plots consisting of four regions. The first region *R*_1_, according to the false positive rate, is between 0 and 0.01; the second region *R*_2_ between 0.01 and 0.1; the third *R*_3_ between 0.1 and 0.23, and the fourth one *R*_4_ with larger values than the previous one (vertical lines Figure 6).

In *R*_1_, all methods express similar behavior and thus it is not fair to rank them. In *R*_2_, the method proposed by Matsushita *et al* has a performance that is superior to the others. In *R*_3_, a mixture of information in the performance of the methods appears, while in *R*_4_ a significant difference between the algorithms is apparent. In general, Figure 6 shows that there are two main tendencies in the efficiency of the methods, one for gray and color Retinex and the other for Weiss and Matsushita *et al* This is due to the fact that these two methods are very similar.

**Figure 7.**
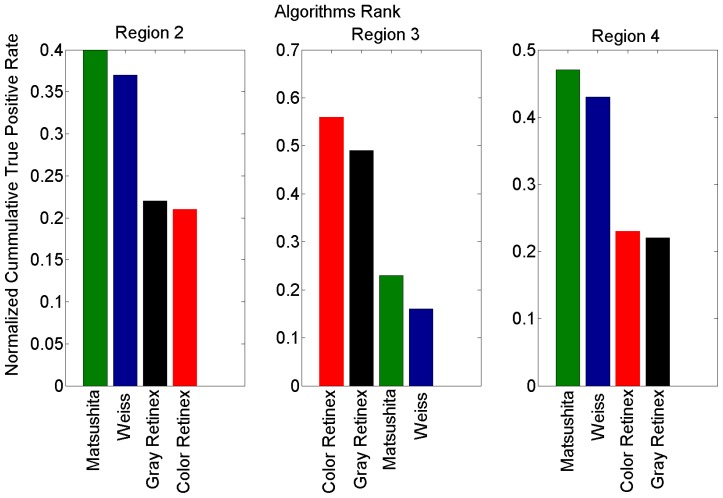
Quantitative comparison of all the algorithms. The regions *R*_2_, *R*_3_ and *R*_4_, correspond to the interval of analysis defined by the vertical lines of Figure 6.

Figure 7 illustrates the ranking of the methods in the four regions of Figure 6. We used the normalized cumulative value of true positive rate (TPR) in each region to rank the methods. Based on these results, we considered the second region to extract and present the reflectance and illumination images in Figures 2 and 4. Moreover, all shadow detection results in Figure 5 were extracted from each respective data set with a 10% value for the false positive rate as the threshold parameter.

### 5.3. Qualitative Analysis

Figure 5 illustrates some shadow regions correctly detected. In the second row, the ground truth of shadows is illustrated. The ground truth of the shadows has been annotated manually to serve as a qualitative comparison between the methods to derive intrinsic images and the detection of shadows.

The Weiss [19] and Matsushita *et al.* [20] methods show better results than the strategies based on the Retinex algorithm [39,40]. The results of the Retinex algorithms applied to the synthetic sequence with white lines on the road surface are less effective than the other methods. The problem with the Retinex algorithm is that it is too constrained. It assumes that small values of the magnitude of the gradient are always due to changes in the illumination of the scene, but in general this idea is not always true.

In the second synthetic sequence, which consists of several static objects in an urban scenario, the methods can detect isolated shadows with good visual performance (see the pine tree and the tower in columns 4–6 in Figure 5). However, other objects such as the house and the trees that appear in the back part of the scene behind the tower, together with the sky line, have poor visual quality. These results are due to the remnant texture information present in the illumination component before the application of the shadow detection process. In the real images of Figure 5, variations in the intensity value of large shaded regions cause the algorithms to only be partially successful. As a result, some regions that have shadows are not well detected. For instance, the region at the right side of the image with the white brick wall (see columns 7–9) is not well detected by any of the discussed methods. The Weiss and Matsushita *et al.* methods fail because these regions are shaded all day, so this causes the shadow information to appear in the reflectance image. Similarly, the gray and color Retinex algorithms fail to detect large shadow areas, because the magnitude of the gradient in the shadow edges is similar to those caused by texture or color.

## 6. Conclusions

A primary goal of many computer vision algorithms is to attenuate the effects caused by shadows. Due to several factors, the problem of shadow detection is a complex and open research field. In this paper, we presented an evaluation of several intrinsic image base methods to detect shadows cast by static objects in outdoor locations.

Although these algorithms were not constructed with the purpose of detecting shadows cast by static objects in the outdoors, we can conclude from the experimental results that the efficiency of intrinsic image methods is relatively poor. Quantitatively, the best method to detect shadows after the intrinsic image components are derived is the algorithm proposed by Matsushita *et al.*, but only if we accept a false positive rate (FPR) between 1% and 10%. Finally, in terms of visual comparison and shadow detection accuracy, we conclude that, if the shadows are isolated, all of the methods can detect them.

Future work will focus on the exploration of alternatives to obtain intrinsic images without the texture information remaining in the illumination component or the shadow information remaining in the reflectance image.
